# Persistent and Circulating *Plasmodium falciparum dhfr* and *dhps* Mutations in Busia County, Western Kenya

**DOI:** 10.3390/pathogens15020233

**Published:** 2026-02-20

**Authors:** Loise Ndung’u, Kelvin Thiong’o, Lewis Karani, Stephen Gitahi, Francis Kimani, Mathew Piero Ngugi, Daniel Kiboi

**Affiliations:** 1 Department of Biochemistry, Jomo Kenyatta University of Agriculture and Technology, Nairobi 00200, Kenya; ndungu.loise@jkuat.ac.ke; 2 Department for Biochemistry, Microbiology and Biotechnology, Kenyatta University, Nairobi 00100, Kenya; piero.mathew@ku.ac.ke; 3 Centre for Biotechnology Research and Development, Kenya Medical Research Institute, Nairobi 00200, Kenya; kvthiongo@kemri.go.ke (K.T.); lkarani84@gmail.com (L.K.); fkimani@kemri.go.ke (F.K.); 4 Department of Natural Sciences, Catholic University of Eastern Africa, Nairobi 00200, Kenya; sgitahi@cuea.edu

**Keywords:** *Plasmodium falciparum*, antifolate resistance, *dhfr*, *dhps*, sulphadoxine-pyrimethamine, Busia County

## Abstract

Malaria in pregnancy remains a major driver of poor maternal and neonatal health outcomes in sub-Saharan Africa. For decades, intermittent preventive treatment in pregnancy (IPTp), with sulphadoxine-pyrimethamine (SP), has mitigated malaria-associated health risks, but concerns have been raised regarding accumulated *Plasmodium falciparum* dihydrofolate reductase (*dhfr*) and dihydropteroate synthase (*dhps*) mutations on the efficacy of SP. Western Kenya, including Busia County, is a high malaria transmission setting where molecular surveillance of *dhfr* and *dhps* mutations remains limited. This study assessed the prevalence and haplotype structure of *dhfr* and *dhps* mutations in *P. falciparum* isolates from Busia County, Kenya. A total of 66 samples of *P. falciparum* isolates collected from patients attending Matayos Sub-County Hospital between November 2024 and January 2025 were analysed. PCR amplification and Sanger sequencing targeted *dhfr* codons C50R, N51I, C59R, S108N/T, I164L, and *dhps* codons I431V, S436A/F, A437G, K540E, A581G, and A613S/T to determine mutation frequencies, haplotypes, and combined *dhps* and *dhfr* haplotype profiles. High frequencies of *dhfr* and *dhps* mutations were observed across the parasite isolates. The most common *dhfr* substitutions included N51I (85.2%) and C59R (75.4%), while S108N (32.8%) and S108T (19.7%) were detected at lower frequencies. *Dhfr* haplotypes identified included N51I + C59R, N51I + C59R + S108N, and a N51I + C59R + S108T + I164L variant. The I164L mutation was detected at a frequency of 18.0% and was observed exclusively on a non-canonical S108T background (19.7%). *Dhps* haplotypes were dominated by A437G (92.3%), K540E (40%) alone, and the A437G + K540E double mutant. Combined *dhfr* and *dhps* haplotype analyses revealed circulation of classical *dhfr* triple-mutant (N51I + C59R + S108N) backgrounds with dhps A437G. Quintuple haplotypes (*dhfr* N51I + C59R + S108T + I164L with *dhps* A437G) and rare complex haplotypes incorporating both I164L and K540E or I164L and S436F were also detected. These findings indicate the persistence and circulation of both canonical and non-canonical *dhfr* and *dhps* haplotypes in *P. falciparum* isolates from Busia County. This study highlights the need for continuous molecular and phenotypic surveillance to clarify the functional and epidemiological significance of parasites carrying S108T and I164L mutations, and to inform IPT policy.

## 1. Introduction

Malaria caused by *Plasmodium falciparum* remains a major public health challenge, with an estimated 263 million cases and 567,000 deaths globally in 2023, the majority occurring in sub-Saharan Africa [1]. Pregnant women and young children bear a disproportionate burden of disease, and *P. falciparum* infection during pregnancy is associated with maternal health risks, including anaemia, morbidity, and mortality, as well as fetal adverse outcomes such as low birth weight, preterm delivery, and miscarriage [2]. To mitigate the fetal adverse outcomes and maternal health risks, the World Health Organization recommends intermittent preventive treatment (IPT) with SP in pregnant women and infants in areas of moderate to high malaria transmission [3,4]. Sulphadoxine-pyrimethamine is a fixed-dose antifolate combination comprising pyrimethamine, which inhibits dihydrofolate reductase (DHFR), and sulphadoxine, which targets dihydropteroate synthase (DHPS), thereby disrupting folate biosynthesis and parasite replication [5]. However, the effectiveness of SP may progressively be compromised by the spread of *P. falciparum* strains carrying resistance-conferring point mutations in the *dhfr* and *dhps* genes [6,7,8]. Canonical *dhfr* mutations (N51I, C59R, S108N) combined with dhps variants (A437G, K540E) form the well-characterized “quintuple mutant” haplotype, which is strongly predictive of SP treatment failure [9,10]. Additional mutations, including *dhps* A581G and *dhfr* I164L, reported in parts of East and West Africa, have been associated with higher-level antifolate resistance and raise concerns about the continued efficacy of SP for IPT [11,12].

Despite near fixation of the *dhfr* triple mutant across much of sub-Saharan Africa, the prevalence and spatial distribution of *dhps* mutations remain heterogeneous, reflecting differences in transmission intensity, drug pressure, and parasite population structure [12,13]. In Kenya, SP was withdrawn as first-line therapy for uncomplicated malaria following the adoption of artemisinin-based combination therapies, yet it remains the recommended drug for IPT, maintaining selective pressure on parasite populations [4,14]. In western Kenya, molecular surveillance studies consistently report high frequencies of *dhfr* and *dhps* resistance alleles, with the quintuple mutant haplotype circulating across diverse transmission settings [12,15]. Previous and more recent reports describe the presence of additional *dhps* mutations, including A581G and S436H, suggesting continued diversification of SP resistance profiles [16,17,18]. However, the prevalence and combinations of *dhfr* and *dhps* mutations vary across geographic regions. Notably, despite the high prevalence of resistance-associated haplotypes, SP for IPT continues to confer partial protection against maternal health risks and adverse birth outcomes, highlighting the complex relationship between molecular resistance markers and clinical efficacy [19]. Despite extensive surveillance elsewhere in western Kenya, data on the composition, co-occurrence, and prevalence of *dhfr* and *dhps* resistance haplotypes in Busia County remain limited. In particular, the occurrence of mutations outside canonical backgrounds and the persistence of low-frequency sextuple mutant haplotypes are poorly characterized. Previous molecular surveillance in two regions of Busia County has documented persistent antifolate resistance-associated *dhfr* and *dhps* mutations, including low-frequency S436H/F/A/Y *dhps* variants and an apparent absence of *dhfr*-A581G, highlighting fine-scale geographic heterogeneity and the importance of local genetic monitoring [20]. Here, we performed molecular mapping of *dhfr* and *dhps* mutations in *P. falciparum* isolates collected in Busia County, western Kenya, providing further refinement of *dhfr*-*dhps* haplotype patterns in this study area. We describe the prevalence, haplotype composition, and co-occurrence patterns of antifolate resistance markers, providing insight into circulating parasite haplotypes in the study population. Although specific *dhfr* and *dhps* mutations have been associated with reduced SP efficacy in other settings, this study did not directly assess clinical or in vivo treatment outcomes, and therefore, functional interpretations of mutations and inferences on IPT are indirect and cautious.

## 2. Materials and Methods Study Site

**Figure 1:**
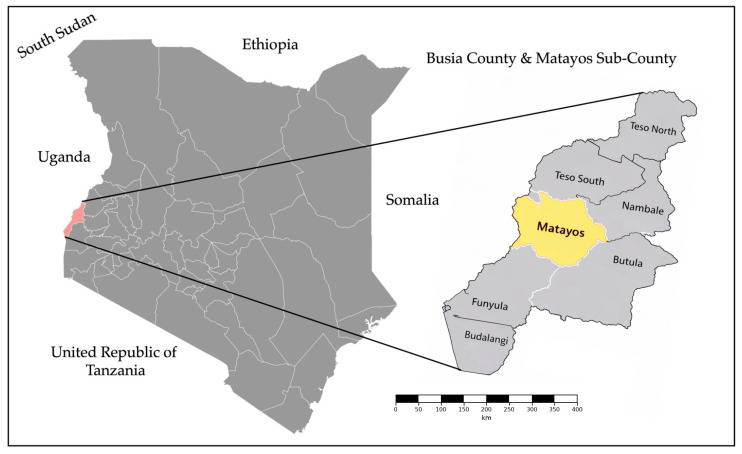
Map of Kenya highlighting Busia County (pink) with an inset showing Matayos Sub-County (yellow), the study site for monitoring *Plasmodium falciparum* malaria infections.

The samples analysed in this study were collected between November 2024 and January 2025 from patients presenting with malaria symptoms at Matayos Sub-County Hospital in Busia County, Western Kenya. The region experiences a high malaria burden with perennial transmission and seasonal peaks following the March–April and November–December rains. Matayos Sub-County is situated in the central-western part of Busia County (Figure 1) and is adjacent to Busia Town, the county headquarters, and its location near the Uganda border facilitates cross-border human movement, which may contribute to local malaria transmission in the area. Due to persistently high malaria incidence, Matayos Sub-County was prioritized for intensified malaria control interventions, including indoor residual spraying (IRS) and larval source management, as part of broader county-wide vector control programs [21].

### 2.1. Recruitment of Study Participants and Sample Collection

Patients aged ≥6 months presenting with malaria symptoms at Matayos Sub-County Hospital were screened for malaria and informed about the study by clinical staff. Interested patients, or, for minors, their parent/guardian, provided informed consent, while children aged 12–17 years provided assent. Pregnant women were excluded from the study. Eligible patients with confirmed *Plasmodium falciparum* mono-infection and parasitaemia between 1000 and 200,000 parasites/µL were assigned a screening number and evaluated by study nurses and clinical officers. Children were assessed for other febrile illnesses (fever > 37.5 °C, chills, sweating, headache, weakness), and those affected were excluded. Clinical assessments included a structured questionnaire capturing recent health history, fever episodes, and antimalarial use in the previous month. Following consent, a finger prick was performed for diagnostic microscopy, and 3 mL of venous blood was collected into BD Vacutainer^®^ ACD Solution A tubes (6 mL) (BD, Franklin Lakes, NJ, USA; Cat. no. 364606) before treatment with artemether-lumefantrine (AL), the standard first-line therapy per Ministry of Health guidelines. From each sample, six dried blood spots (≈50 µL each) were prepared on NucleoCard^®^ blood sample storage card, (MACHEREY-NAGEL, Düren, Germany, Cat. no. 740403.100), air-dried, individually sealed with silica gel desiccant, and stored at room temperature. Samples were subsequently transported to the JKUAT-SAJOREC laboratory for molecular analysis.

### 2.2. DNA Extraction

Genomic DNA was extracted from dried blood spots (DBS) using the QIAamp DNA Mini Kit (QIAGEN, Hilden, Germany, Cat. no. 51306) following the manufacturer’s protocol with minor modifications to optimize yield. For each sample, two to three 3 mm punches were collected using sterile forceps, sampling from both the center and periphery of the DBS card to ensure representative parasite recovery. The punches were incubated in ATL buffer and Proteinase K at 56 °C for approximately 60 min, or until complete lysis was visually confirmed. Following lysis, ethanol was added, and samples were transferred to QIAamp spin columns for DNA binding and purification. Genomic DNA was eluted in 100 µL of AE buffer and stored at −20 °C until PCR amplification and downstream molecular analyses.

### 2.3. PCR Amplification of the dhfr and dhps Target Regions

Gene-specific primers were designed to amplify regions of the *dhfr* and *dhps* genes encompassing key codons previously associated with antifolate resistance, namely *dhfr* codons 50, 51, 59, 108, and 164, and *dhps* codons 431, 436, 437, 540, 581, and 613 (Table 1). PCR amplification was performed in a total reaction volume of 20 µL, comprising 10 µL of Platinum™ SuperFi II Green PCR Master Mix (2X) (Invitrogen™, Thermo Fisher Scientific, Waltham, MA, USA; Cat. no. 12369050), 2 µL of genomic DNA template, 1 µL of each forward and reverse primer, and 6 µL of nuclease-free water. Thermal cycling was conducted using the conditions detailed in Table 1, optimized to ensure high-fidelity amplification of each target region. PCR products were verified by electrophoresis on a 1.5% agarose gel stained with SYBR Safe DNA Gel Stain (Thermo Fisher Scientific™, Cat. No. S33102, Waltham, MA, USA) and visualized under UV illumination to confirm the expected amplicon size. Successfully amplified products were purified using the QIAquick PCR Purification Kit (QIAGEN, Hilden, Germany, Cat. no. 28106) according to the manufacturer’s protocol. Purified amplicons were then subjected to bidirectional sequencing using the BigDye^®^ Terminator v3.1 Cycle Sequencing Kit (Applied Biosystems™, Thermo Fisher Scientific, Waltham, MA, USA; Cat. no. 4337455) and resolved on an ABI 3730xl DNA Analyzer (Applied Biosystems™, Thermo Fisher Scientific, Waltham, MA, USA) to generate high-quality sequence reads for downstream analysis.

### 2.4. Sequence Analysis and Variant Calling

Raw Sanger chromatogram sequences for the *P. falciparum dhfr* and *dhps* genes were first imported into Geneious Prime^®^ 2025.2 (Biomatters Ltd., Auckland, New Zealand) for quality control. All chromatograms were inspected for evidence of mixed infections or heterozygous peaks; however, due to the limited sensitivity of capillary-based Sanger sequencing in resolving and detecting low-frequency variants in polyclonal infections, potential minor alleles could not be reliably assessed and were excluded from the analysis. Low-quality nucleotide calls were trimmed using the default Geneious trimming algorithm with an error probability limit of 0.05, and all sequences were visually inspected to confirm the accuracy of base calling. Following trimming, the cleaned sequences were exported as CSV files and analysed in RStudio (R version 4.5.1). Within R, sequences were preprocessed by removing ambiguous bases, filtering out reads containing more than 5% unresolved nucleotides, and standardizing sequence formats using functions from the Biostrings, seqinr, and tidyverse packages. Reference sequences for *dhfr* (PF3D7_0417200) and *dhps* (PF3D7_0810800) were obtained from PlasmoDB release 68 (May 2024) and used as alignment templates. Multiple sequence alignments were performed in R using the CLUSTALW algorithm implemented in the msa package, and all alignments were inspected to ensure correct codon alignment and the absence of frame shifts or spurious gaps. Variant calling was conducted by comparing each sample sequence to the *Plasmodium falciparum* 3D7 reference using custom R scripts built on the Biostrings and DECIPHER frameworks, allowing systematic identification of both synonymous and nonsynonymous single-nucleotide polymorphisms. Codons were translated in frame to annotate amino acid substitutions following standard nomenclature, and all detected mutations were tabulated alongside their frequencies and known associations with antifolate resistance based on published literature and PlasmoDB annotations.

### 2.5. Ethical Considerations

This study received ethical approval from the Scientific and Ethics Review Unit (SERU) (KEMRI/SERU/CBRD/267/5005) of the Kenya Medical Research Institute (KEMRI), Busia, County Health Management Committee (CHMT), and a research license was granted by the National Commission for Science, Technology, and Innovation (NACOSTI) (NACOSTI/P/25/414446). Written informed consent was obtained from all adult participants, and assent, together with parental or guardian consent, was obtained for participants under 18 years of age. All study procedures, including sample collection, data handling, and laboratory analyses, were performed in accordance with institutional guidelines and international standards for good clinical and laboratory practice, ensuring the confidentiality, rights, and welfare of all participants.

## 3. Results

### 3.1. Demographics of Study Participants

A total of 66 participants who met the eligibility criteria were enrolled in the study, comprising 35 males (53.0%) and 31 females (47.0%). Participant ages ranged from 2.7 to 51 years, with the majority of individuals being children and adolescents. Age data were unavailable for five participants. Pregnant women were excluded from enrolment. A summary of participant demographic characteristics is provided in Table 2.

### 3.2. Prevalence of P. falciparum dhfr Mutations and Haplotype Distribution

**Figure 2:**
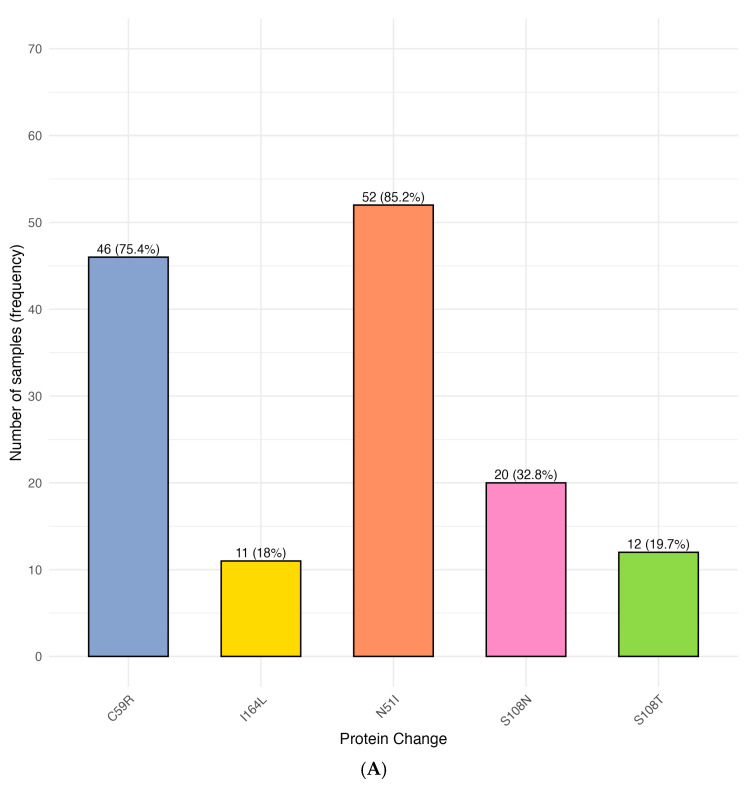

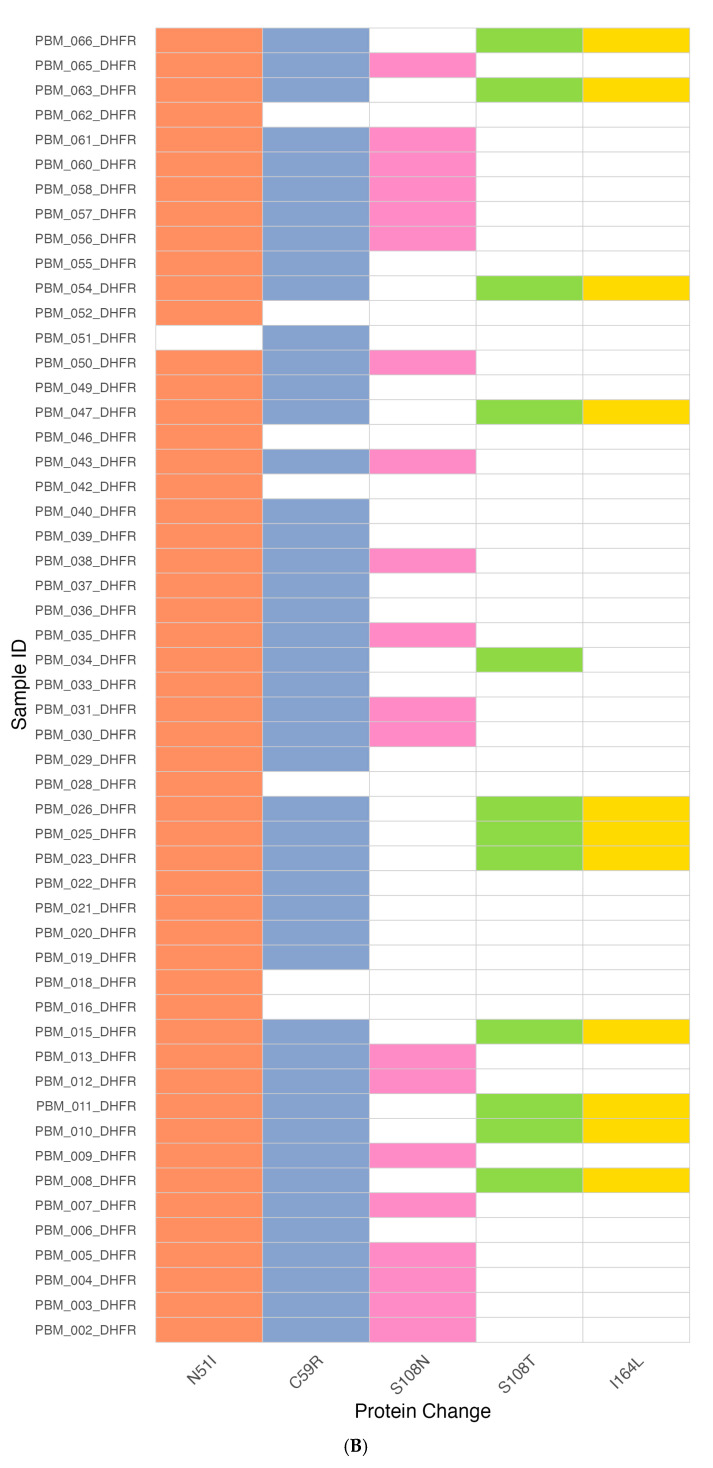
*Plasmodium falciparum* dihydrofolate reductase (*dhfr*) mutations associated with pyrimethamine resistance in parasite isolates from Busia County, Kenya. (**A**) Frequencies of *dhfr* mutations. (**B**) Distribution and co-occurrence of key *dhfr* codons.

We assessed parasite population structure based on the *dhfr* gene and focused first on the five canonical codons and SNPs linked with antifolate-resistance, these are N51, C59, and S108, as well as the less common variants at codons 50 and 164. The prevalence of individual *dhfr* alleles, along with their associated resistance phenotypes, is summarized in Table 3. We reveal a unique S108T that concurrently occurred with the I164L mutation alongside the mutation in codons 51 and 59. Across the study population, the most prevalent change occurred at codon 51, where 52/61 infections harboured the N51I mutation (85.2%) (Figure 2A, Table 3). This mutation seemed to frequently appear in the backdrop of other *dhfr* substitutions. At codon 59, the C59R allele was detected in 46/61 infections (75.4%), consistent with the circulation of well-established antifolate-resistant haplotypes in the region. At codon 108, the S108N substitution was detected in 20/61 parasite isolates, corresponding to 32.8%. Notably, an additional 12 isolates (19.7%) carried the uncommon S108T variant, yielding a combined prevalence of 52.5% for changes at this position. Mutations outside the classical triad were also assessed. No substitutions were observed at codon 50. However, the I164L mutation, typically rare in African *P. falciparum* populations, was detected in 11/61 isolates (18.0%) (Figure 2A). After constructing the common haplotypes (Figure 2B), we show that the N51I/C59R/S108N triple mutant occurred in 20/61 infections (32.8%). Double-mutant combinations were common (Figure 2B and Figure S1A). N51I + C59R was present in 45 isolates (73.8%), while N51I + S108N and C59R + S108N each appeared in 20 infections (32.8%). Parasite isolates harbouring N51I + I164L or C59R + I164L were identified in 11 cases each (18.0%), whereas no sample showed the S108N + I164L combination. The classical quadruple mutant (N51I/C59R/S108N/I164L) was not observed. Surprisingly, a distinct haplotype comprising N51I, C59R, S108T, and I164L was identified in 12/61 infections (19.7%), with I164L only appearing in the background on S108T alongside the recognized (N51I + C59R) resistance markers.

### 3.3. Prevalence of *P. falciparum dhps* Mutations and Haplotype Distribution

**Figure 3:**
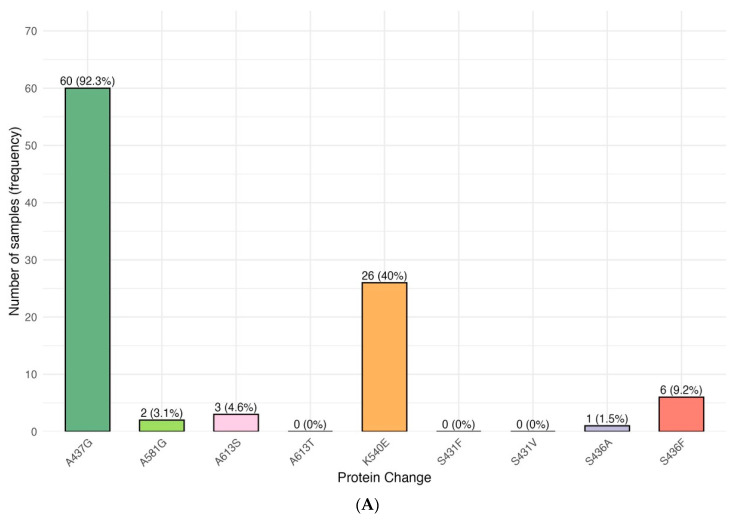

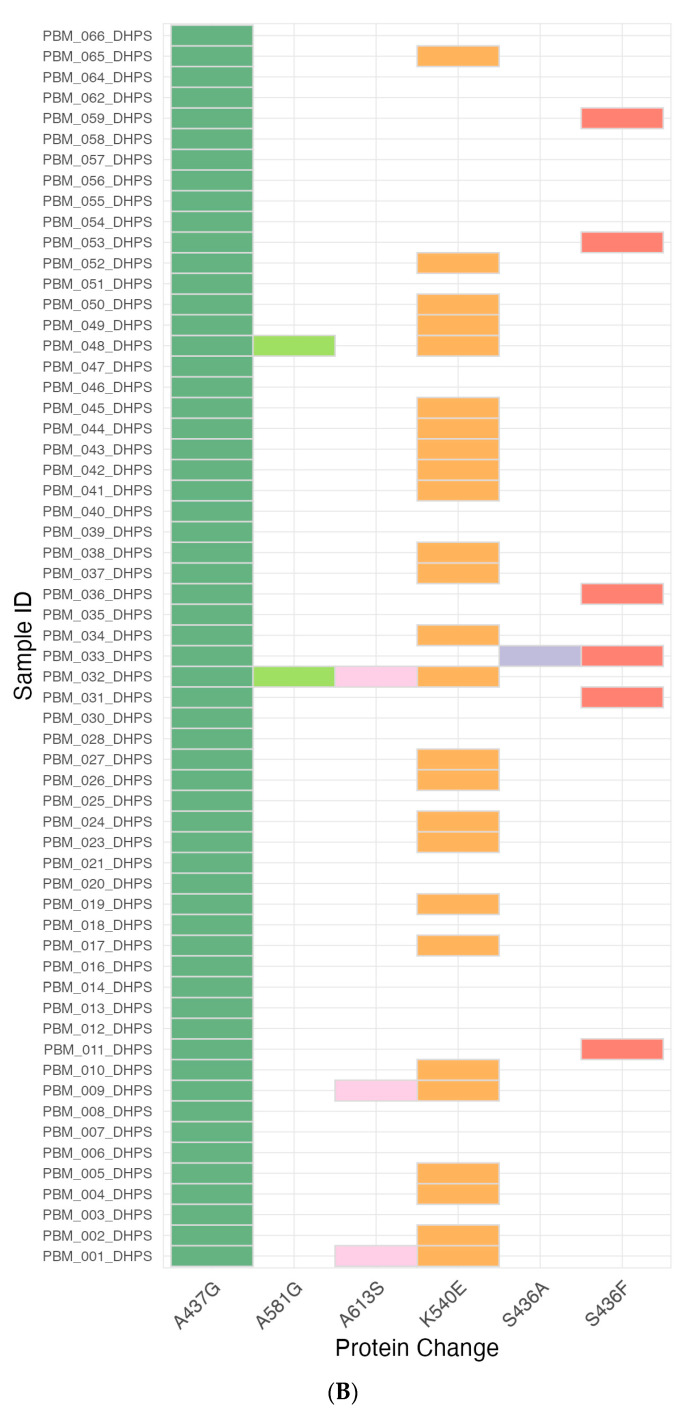
*Plasmodium falciparum* dihydropteroate synthase (*dhps*) mutations associated with sulphadoxine resistance in parasite isolates from Busia County, Kenya. (**A**) Frequencies of *dhps* mutations. (**B**) Distribution and co-occurrence of key *dhps* codons.

To deconvolute the sulphadoxine resistance markers in the clinical samples, we focused on known and new non-synonymous single-nucleotide polymorphisms (SNPs) at established *dhps* codons (436, 437, 540, 581, and 613). The distribution of *dhps* alleles and their inferred sulphadoxine resistance profiles are presented in Table 3. Of the 66 *Plasmodium falciparum*–positive clinical samples collected, 65 yielded high-quality *dhps* sequences and were included in the analysis. The frequency distribution of individual mutations is shown in Figure 3A. The A437G mutation was the most prevalent, detected in 60 of 65 isolates (92.3%). The K540E mutation was identified in 26 isolates (40.0%) (Table 3). Mutations at codon 436 occurred at lower frequencies, with S436F detected in 6 isolates (9.2%) and S436A in 1 isolate (1.5%). Low-frequency mutations were also observed at codons 581 and 613, with A581G present in 2 isolates (3.1%) and A613S in 3 isolates (4.6%). No non-synonymous substitutions were detected at codons S431 or A613T. Haplotype analysis revealed multiple combinations of *dhps* mutations (Figure 3B and Figure S1B). The A437G + K540E double-mutant haplotype was the most common, occurring in 22 of 65 isolates (33.8%). Triple-mutant haplotypes were uncommon: one isolate carried A437G + K540E + A581G (1.5%), and two isolates carried A437G + K540E + A613S (3.1%). A single isolate (1.5%) harboured a quadruple-mutant haplotype comprising A437G + K540E + A581G + A613S (Figure S1B). No other quadruple-mutant combinations were observed. Among isolates carrying at least one frequently observed *dhps* mutation, A437G was present in all cases (100%).

### 3.4. Combined Frequencies of dhfr-dhps Haplotypes

**Figure 4:**
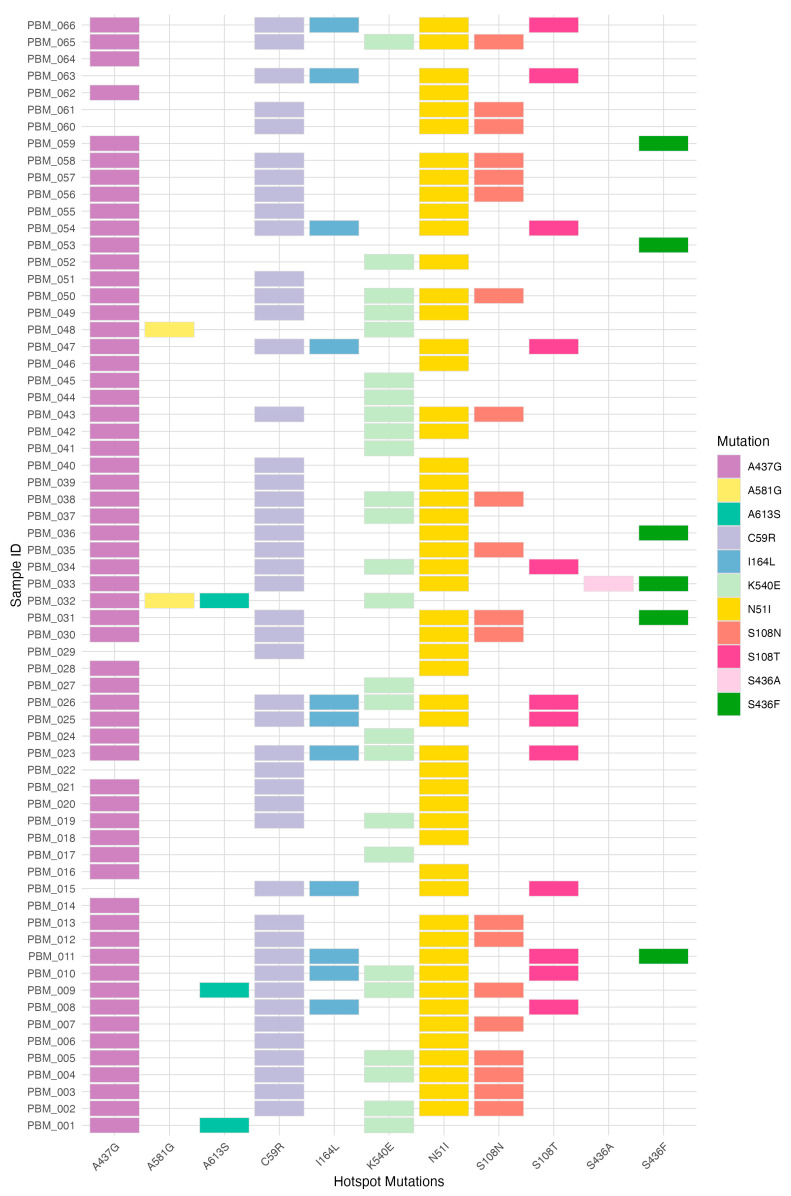
Heatmap of *P. falciparum dhfr* and *dhps* mutations illustrating the distribution and co-occurrence of pyrimethamine-sulphadoxine resistance haplotypes.

We next examined combined haplotypes spanning both *dhfr* and *dhps* to assess the co-occurrence of antifolate resistance markers within individual infections. Analysis of combined *dhfr-dhps* haplotypes showed variation in haplotype composition, including the detection of high antifolate resistance genotypes incorporating the *dhfr* I164L mutation (Figure 4 and Figure S2). The most prevalent haplotype was *dhfr-*N51I + C59R + S108N-*dhps-*A437G, identified in 10 isolates (21.3%), a classical *dhfr* triple-mutant background combined with *dhps* A437G. The second most frequent haplotype was *dhfr-*N51I + C59R-*dhps-*A437G (8 isolates; 17.0%), followed by *dhfr-*N51I + C59R + S108N-*dhps-*A437G + K540E (7 isolates; 14.9%), genotypes harbouring both the *dhfr* triple mutations and the key *dhps* K540E substitution within the sampled population. Notably, I164L-containing haplotypes were detected at appreciable frequencies. The quintuple-mutant *dhfr* haplotype *dhfr*-N51I + C59R + S108T + I164L-*dhps*-A437G was observed in 6 isolates (12.8%), while the corresponding composite mutant haplotype incorporating *dhps* K540E (*dhfr-*N51I + C59R + S108T + I164L-*dhps-*A437G + K540E) was present in 3 isolates (6.4%) (Figure 4 and Figure S2). Intermediate frequency haplotypes included *dhfr-*N51I-*dhps-*A437G (5 isolates; 10.6%) and *dhfr-*N51I + C59R-*dhps-*A437G + K540E (3 isolates; 6.4%). Less frequent haplotypes included *dhfr-*N51I-*dhps-*A437G + K540E (2 isolates; 4.3%) and rare genotypes as *dhfr-*C59R-*dhps-*A437G, *dhfr-*N51I + C59R + S108N-*dhps-*A437G + K540E + A613S, and *dhfr-*N51I + C59R + S108T-*dhps-*A437G + K540E, each was detected in a single isolate (2.1% each).

## 4. Discussion

Mutations in the *dhfr* and *dhps* genes remain central markers of SP resistance and are widely used to monitor the distribution of antifolate-resistant *Plasmodium falciparum* globally [5,22]. In East Africa and Kenya in particular, the deployment of SP as first-line therapy in the late 1990s [14] introduced pressure on parasite populations, potentially driving the occurrence of the canonical *dhfr* triple-mutant haplotype N51I/C59R/S108N, which is strongly associated with high-level pyrimethamine resistance [9,10]. Using integrated single-locus and combined *dhfr-dhps* haplotype analyses, our findings indicate that the parasite population from Matayos, Busia County, Kenya, is characterized by a substantial presence of well-established canonical antifolate resistance alleles, alongside the detection of the atypical *dhfr* I164L mutation occurring within a non-canonical genetic background. The high prevalence of *dhfr* N51I (85.2%) and C59R (75.4%) mutations likely reflects historical antifolate drug selection and is consistent with patterns reported across Western Kenya and the broader East African region [13,15,23]. The occurrence of non-S108N haplotypes carrying N51I and C59R is rare but does occur [12]. These N51I + C59R mutations typically occur on an S108N-containing background, which is the primary determinant of pyrimethamine resistance. The additional detection of I164L alongside established resistance markers has been associated in other studies with higher pyrimethamine resistance, although this was not directly assessed in our study population. The persistence of these canonical *dhfr* resistance alleles more than two decades after withdrawal of SP for routine case management [4,14,24] may reflect their minimal fitness cost in the mutant parasites.

Studies from Western Kenya have demonstrated sustained fixation of *dhfr* resistance markers in both pregnant women and asymptomatic infections, reinforcing the concept that these mutations are now deeply entrenched within local parasite populations [17,23]. At a continental scale, recent genomic surveillance similarly confirms that *dhfr* resistance alleles have been entrenched at stable prevalence levels in many African settings, even where SP use has substantially declined [12,13]. In this study, the frequent co-occurrence of *dhfr* alleles within double- and triple-mutant haplotypes further suggests that these resistance determinants are now deeply embedded and freely circulating within the local parasite gene pool. Beyond these well-established patterns, our data reveal notable diversification within *dhfr*, particularly at codon 108, with a combined frequency of 52.5% when both S108N and S108T variants were considered. While S108N remains, the canonical mutation associated with pyrimethamine resistance, the relatively high frequency of the less common S108T variant is notable and may reflect localized circulation of distinct parasite genotypes. Importantly, S108T was not observed in isolation but consistently occurred in combination with other resistance-associated mutations, most notably the resistance mutation, I164L, implying a possible functional relevance rather than neutral polymorphism.

The detection of *dhfr* I164L in 18% of isolates represents one of the most significant findings of this study. Historically, the I164L mutation has been rare or absent in African *P. falciparum* populations and is primarily reported in Southeast Asia and South America, where it has been associated with high-level antifolate resistance [25,26,27]. Interestingly, I164L was not observed in combination with the classical founder S108N and African quadruple-mutant background (N51I/C59R/S108N/I164L). Instead, I164L appeared exclusively in a distinct genetic background comprising N51I, C59R, and the uncommon S108T substitution. The detection of the N51I + C59R + S108T + I164L haplotype in nearly 20% of infections highlights the presence of a non-canonical *dhfr* haplotype within the study population, differing from resistance patterns commonly reported in other African regions, the functional significance of which remains to be determined. This pattern may reflect compensatory or epistatic interactions between S108T and I164L that permit parasite viability while conferring high-level resistance. The absence of S108N + I164L combinations highlights distinct *dhfr* haplotype patterns in the parasite population, and certain mutational combinations may be uncommon in the study area.

Complementing the *dhfr* profile, mutations in the *dhps* gene play a critical role in sulphadoxine resistance and in determining the overall efficacy of SP-based interventions [22]. We equally note high prevalence of *dhps* resistance markers that may have implications for sulphadoxine efficacy. Consistent with reports from East Africa, the dhps A437G mutation was highly prevalent in our samples (92.3%), reflecting its well-established role as a key mutation associated with sulphadoxine resistance. [9,11,12,23]. The prevalence of K540E (40.0%) indicates that a notable fraction of parasites carries this key dhps mutation, although full fixation of this mutation may not yet have occurred in the population. Our findings align with evidence of marked spatial heterogeneity in its distribution across Western Kenya and along the Kenya-Uganda border [15,17] and contrast with reports of very low or absent K540E frequencies in other areas of Busia County [20]. In addition to classical East African alleles, we detected several other *dhps* mutations, including S436F, S436A, A581G, and A613S/T. While some of these mutations have historically been more prevalent in West Africa or Southeast Asia [12,28], growing evidence indicates increasing geographic intermixing of antifolate resistance haplotypes across Africa [12,18,28]. Similar diversification of *dhps* genotypes has been reported in Western Kenya using both conventional and deep sequencing approaches [15,16,29]. Of particular concern is the detection of A581G, which has been linked to reduced IPT-SP efficacy and adverse pregnancy outcomes, including low birth weight and placental malaria [30,31,32].

Analysis of combined *dhfr-dhps* haplotypes provides a more comprehensive forecast of antifolate resistance than single-gene analyses alone [10,31,33]. The predominance of haplotypes combining *dhfr* triple mutants with *dhps* A437G and, to a lesser extent, K540E underscores the presence of parasites associated with reduced susceptibility to both pyrimethamine and sulphadoxine. Of particular concern is the identification of combined haplotypes incorporating *dhfr* I164L together with *dhps* K540E, which represent high-order resistance genotypes rarely reported in African settings. Notably, the detection of a composite mutant haplotype (*dhfr* N51I + C59R + S108T + I164L combined with *dhps* A437G + K540E) and *dhfr* N51I + C59R + S108T + I164L + with *dhps* S436F+A437G represents a genotype previously associated with high levels of antifolate resistance in other studies. The presence of these haplotypes, even at moderate frequencies, suggests the presence of parasite genotypes historically associated with near-complete SP treatment failure. Overall, our findings may have implications for malaria control and surveillance indicating that *dhfr* mutations occur at relatively high frequencies in the local parasite population, whereas *dhps* mutations show heterogeneous frequency patterns. The identification of a *dhfr* I164L-containing haplotype underscores the importance of fine-scale molecular surveillance capable of detecting non-canonical resistance pathways before they become widespread. Given the observed prevalence of parasites carrying the S108T and I164L mutations, we recommend follow-up studies combining phenotypic antifolate susceptibility assays, expanded multi-site surveillance, and genetic background analyses to determine the functional relevance and epidemiological significance of parasites carrying the S108T and I164L mutations. Such studies will help safeguard SP-based preventive interventions, inform IPT policy, and guide targeted malaria control strategies.

A limitation of this study is the relatively small sample size of 66 isolates, which corresponds to patients who tested positive for *P. falciparum* and met the eligibility criteria despite Busia County being a high malaria transmission setting. The lower-than-expected sample number likely reflects the impact of ongoing malaria control interventions, such as insecticide-treated nets (ITNs), indoor residual spraying (IRS) supported by the President’s Malaria Initiative [21], and possibly the deployment of the RTS,S malaria vaccine [34], which may have reduced parasite prevalence. Despite the modest sample size, the collected isolates provide a clear snapshot of the circulating *dhfr* and *dhps* haplotypes among the confirmed infections. While larger-scale surveillance would be needed to capture the full range of haplotypes in the region, the current data offer valuable insights into the composition and co-occurrence of antifolate resistance markers within the study population.

## Figures and Tables

**Table 1:** Primer sequences, annealing positions, and PCR amplification conditions used for the amplification and sequencing of *Plasmodium falciparum* dihydrofolate reductase (*Pfdhfr;* PF3D7_0417200) and dihydropteroate synthase (*Pfdhps;* PF3D7_0810800) genes.

Accession Number	Primer Name	PCR and Sequencing Primer Sequence 5′–3′	Primer Annealing Positions
PF3D7_0417200	*Pfdhfr*-forward	CATATGTGCATGTTGTAAGGTTGA	50–64
	*Pfdhfr*-reverse	GCTAACAGAAATAATTTGATACTCA	
PF3D7_0810800	*Pfdhps*-forward	CAGATGGAGGTATTTTTGTTGAAC	431–613
	*Pfdhps*-reverse	GATCCTTGTCTTTCCTCATGT	
PCR amplifying profiles			
		*Pfdhfr*	*Pfdhps*
Initial denaturation		98 °C, 30 s	98 °C, 30 s
Denaturation		98 °C, 10 s	98 °C, 10 s
Annealing		51 °C, 10 s	60 °C, 10 s
Elongation		72 °C, 30 s	72 °C, 30 s
Final Elongation		72 °C, 5 min	72 °C, 5 min
Superfi Master mix		10 μL	10 μL
Cycles		35	35
PCR H_2_O		6 μL	6 μL
Primer (Forward and Reverse)		1 μL each	1 μL each
DNA Template		2 μL	2 μL

**Table 2:** Demographic and enrolment characteristics of study participants.

Patient Characteristics	Value
Total number of participants	Sixty-six (66)
Sex, n (%)	Male: 35 (53%)
	Female: 31 (47%)
Age range, years	2.7–51
Age distribution, n (%)	<5 years: 12 (18.2%)
	5–14 years: 39 (59.1%)
	15–17 years: 4 (6.1%)
	≥18 years: 6 (9.1%)
Age missing, n (%)	5 (7.6%)
Pregnant women	Excluded

**Table 3:** Prevalence of *Plasmodium falciparum* dihydrofolate reductase (*Pfdhfr*) and dihydropteroate synthase (*Pfdhps*) Alleles and Associated Resistance Phenotypes in *P. falciparum* parasite isolates from Busia County, Kenya.

Gene	Mutation	Allele	Phenotype	Frequency (%, n/N)
*Pfdhfr*	C50R	C50	WT	100, (61/61)
		50R	MT	0, (0/61)
	N51I	N51	WT	14.8, (9/61)
		51I	MT	85.2, (52/61)
	C59R	C59	WT	24.6, (15/61)
		59R	MT	75.6, (46/61)
	S108N/T	S108	WT	47.5 (29/61)
		108N	MT	37.8, (20/61)
		108T	MT	19.7 (12/61)
	I164L	I164	WT	82, (50/61)
		164L	MT	18, (11/61)
*Pfdhps*	S431V	S431	WT	100, (65/65)
		431V	MT	0, (0/65)
	S436F/A	S436	WT	89.23 (58/65)
		436F	MT	9.23, (6/65)
		436A	MT	1.54 (1/65)
	A437G	A437	WT	7.7, (5/65)
		437G	MT	92.3, (60/65)
	K540E	K540	WT	60, (39/65)
		540E	MT	40, (26/65)
	A581G	A581	WT	96.9, (63/65)
		581G	MT	3.08, (2/65)
	A613S/T	A613	WT	95.4, (62/65)
		613S	MT	4.6, (3/65)
		613T	MT	0 (0/65)

WT—wild-type allele; MT—mutant allele. Frequency (%, n/N) indicates the percentage and the number of isolates carrying the specified allele out of the total number tested.

## Data Availability

The original data presented in the study are openly available in Zenodo at https://doi.org/10.5281/zenodo.18105538 accessed on 31 December 2025.

## Supplementary Materials

The following supporting information can be downloaded at: https://www.mdpi.com/article/10.3390/pathogens15020233/s1, Figure S1: Frequencies of pyrimethamine-sulphadoxine resistance haplotypes. (A) *Plasmodium falciparum* dihydrofolate reductase (*dhfr*). (B) *Plasmodium falciparum* dihydropteroate synthase (*dhps*), highlighting the co-occurrence of mutations that define antifolate resistance profiles; Figure S2: Frequencies of combined *Plasmodium falciparum* dihydrofolate reductase (*dhfr*) and *Plasmodium falciparum* dihydropteroate synthase (*dhps*) resistance haplotypes in *P. falciparum* parasite isolates from Busia County, Kenya.
