# Supplementary figures and images for: Persistent and Circulating Plasmodium falciparum dhfr and dhps Mutations in Busia County, Western Kenya
Source: Pathogens. 2026 Feb 20;15(2):233. doi: 10.3390/pathogens15020233 (PMC12943605; doi:10.3390/pathogens15020233)

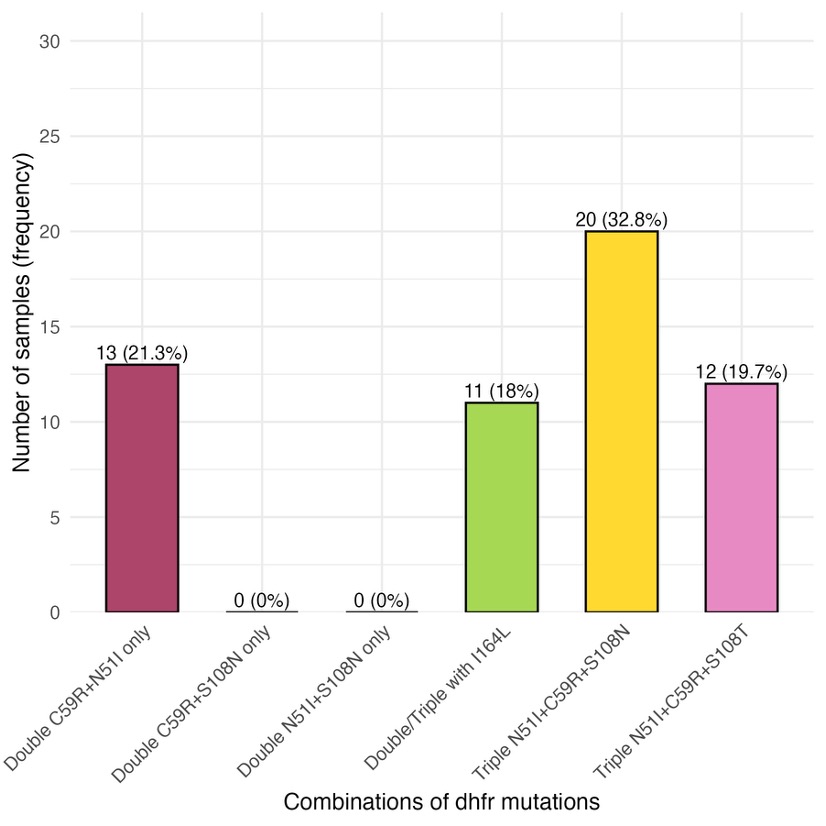

Supplement: Supplementary file 1 [file pathogens-15-00233-s001.zip › Fig. S1A.tiff]

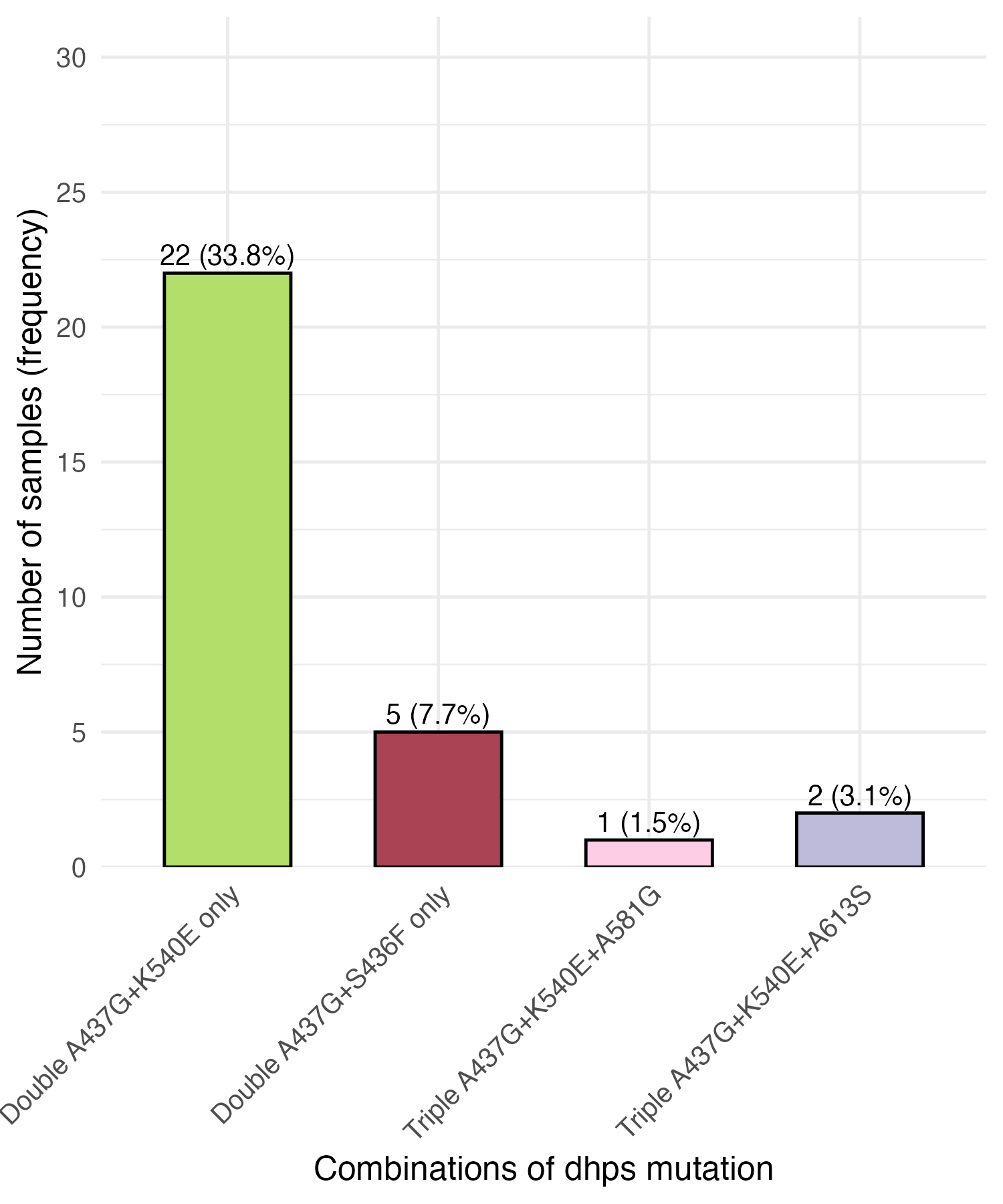

Supplement: Supplementary file 1 [file pathogens-15-00233-s001.zip › Fig. S1B.tiff]

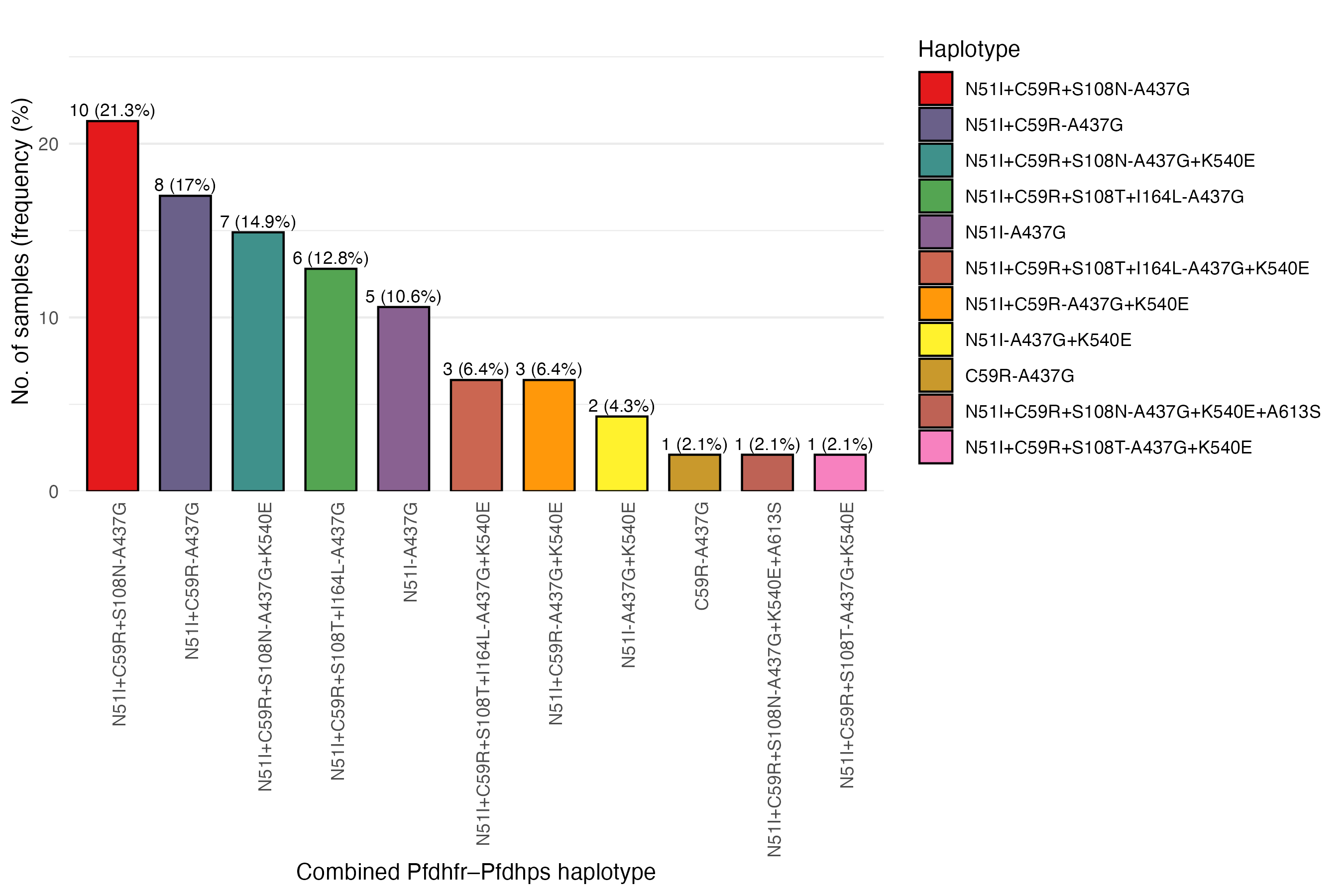

Supplement: Supplementary file 1 [file pathogens-15-00233-s001.zip › Fig. S2.tiff]

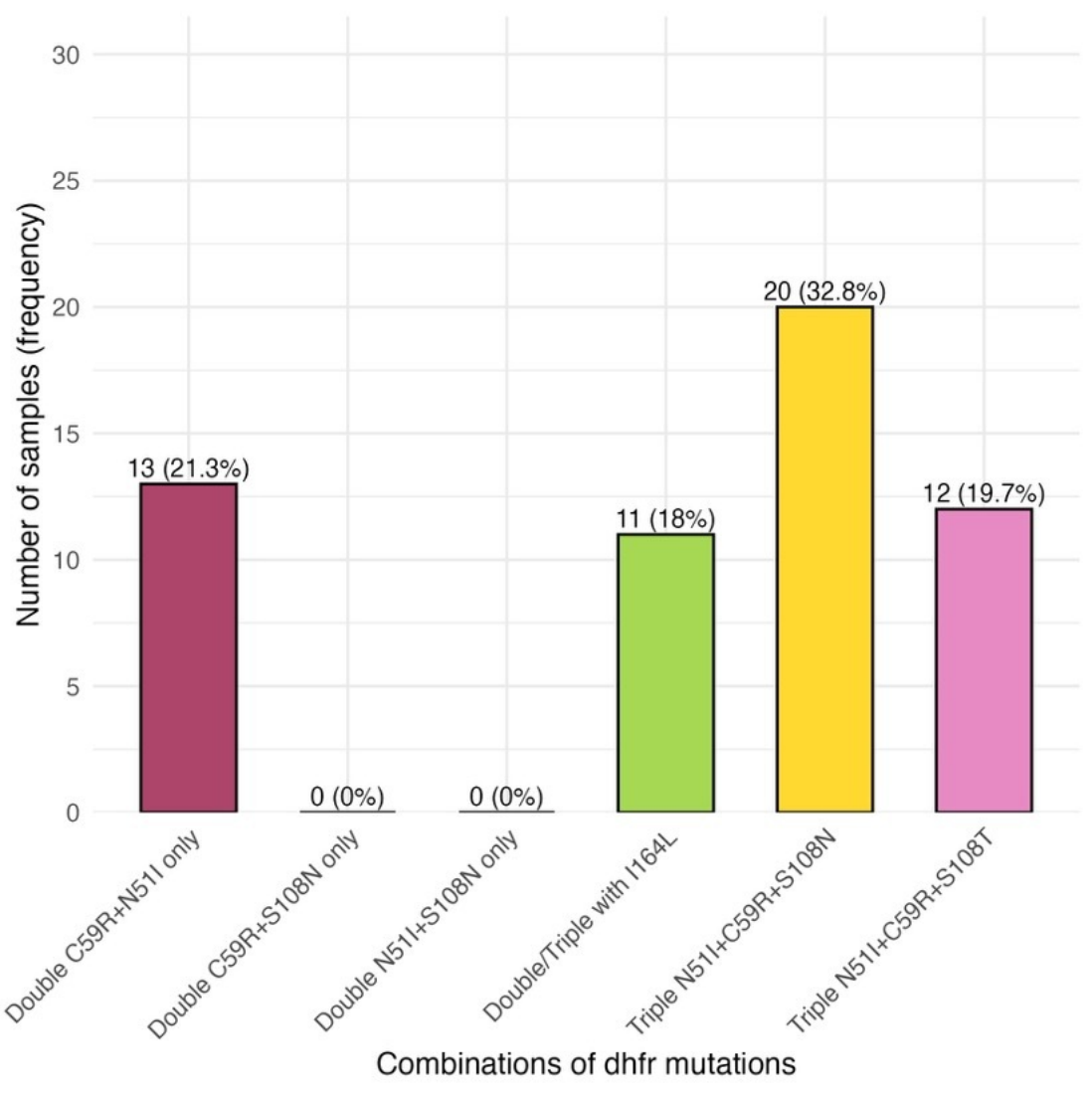

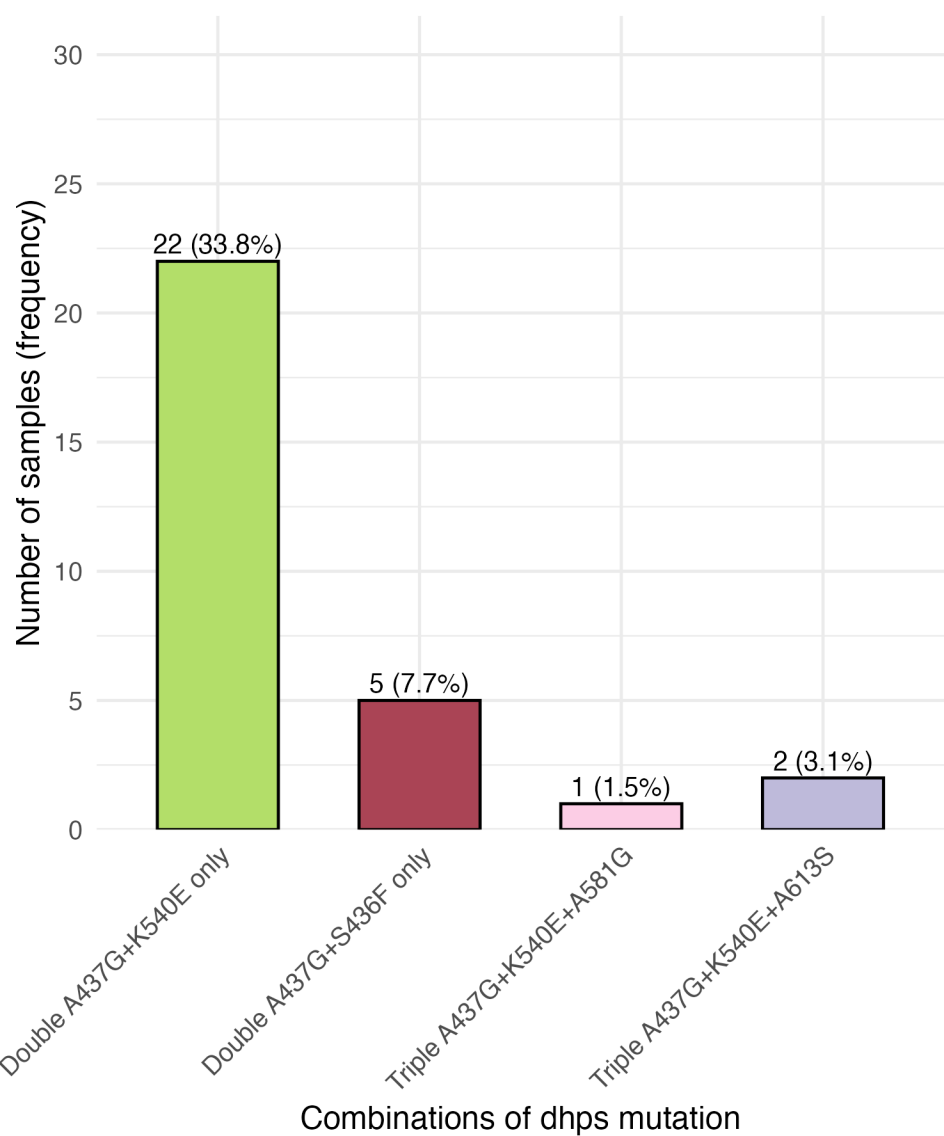

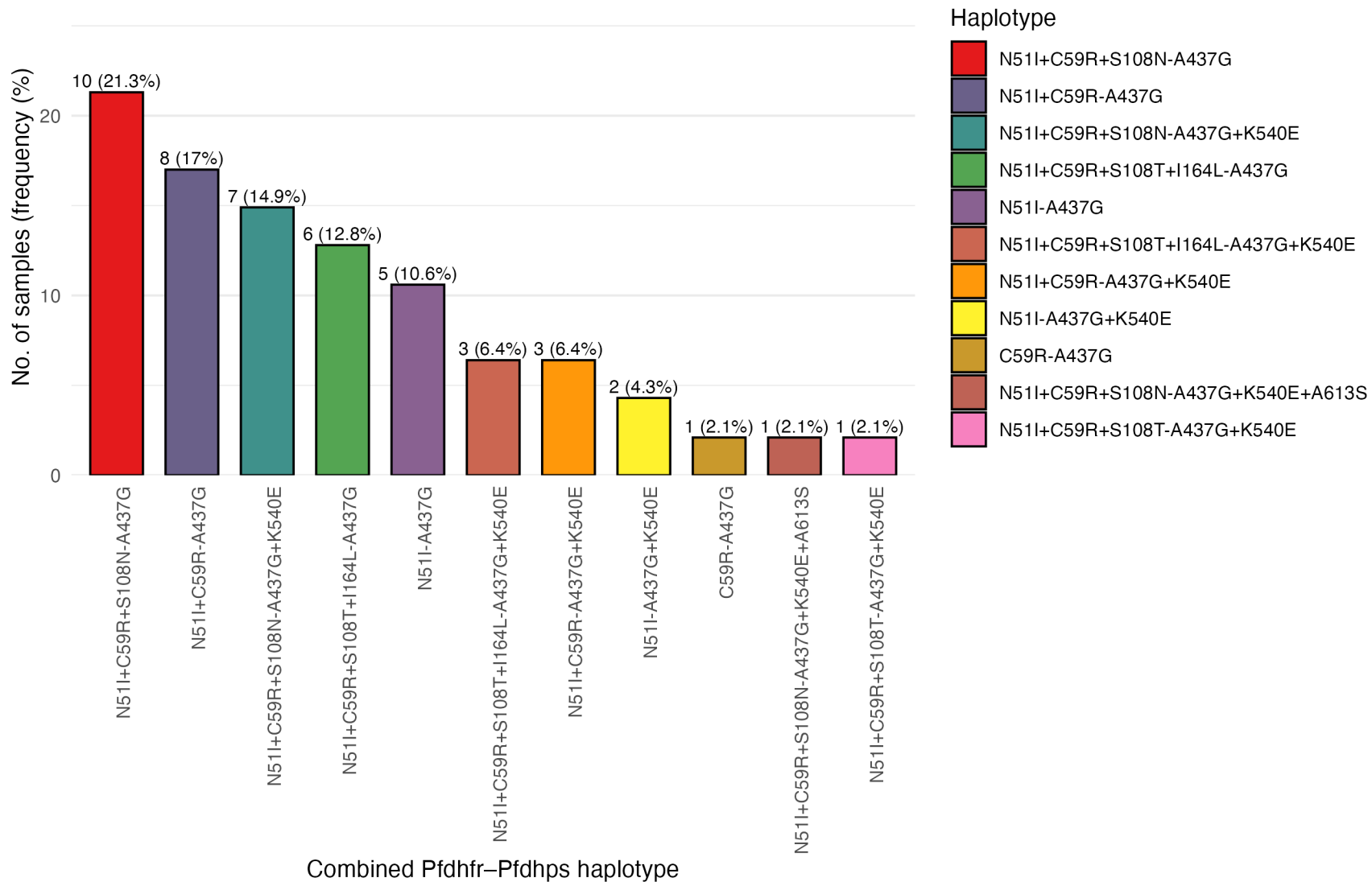

Supplement: Supplementary file 1 [file pathogens-15-00233-s001.zip › merged_output.pdf]

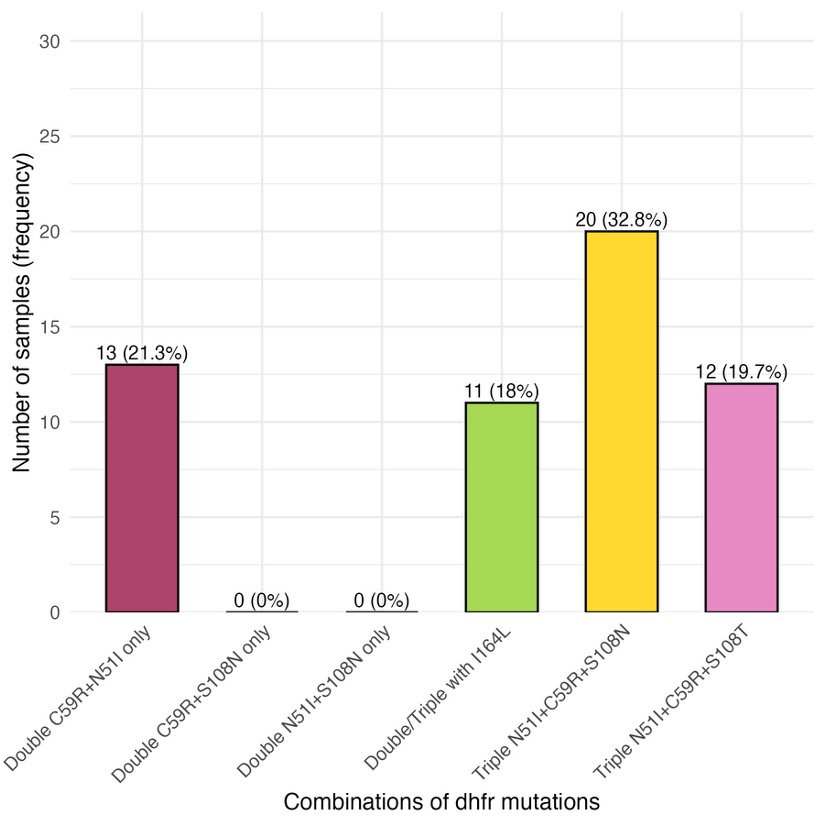

Supplement: Supplementary file 1 [file pathogens-15-00233-s001.zip › supplemental_files.tiff]
